# Self-reported predictors of depressive symptomatology in an elderly population with type 2 diabetes mellitus: a prospective cohort study

**DOI:** 10.1186/1477-7525-5-50

**Published:** 2007-08-02

**Authors:** Manjiri D Pawaskar, Roger T Anderson, Rajesh Balkrishnan

**Affiliations:** 1 Department of Pharmacy Practice and Administration, Ohio State University College of Pharmacy, Columbus, OH, 43210, USA; 2 Wake Forest University School of Medicine, Medical Center Boulevard, Winston-Salem, NC 27157, USA

## Abstract

**Background:**

The prevalence of depression increases among the elderly with chronic medical conditions like diabetes. Hence, the purpose of this study was to determine predictors of depressive symptomatology in Medicare enrolled elderly population with type 2 diabetes mellitus.

**Methods:**

A prospective cohort study was conducted by administrating health risk assessment questionnaire to elderly (≥65 years) with type 2 diabetes. Responses were linked with administrative claim's data. Data were obtained from elderly with type 2 diabetes who were enrolled in Medicare Health Maintenance Organization (HMO) in southeastern United States. The instrument collected information related to demographics, health status, medication use, and healthcare service utilization prior to enrollment. Responses were combined with the administrative claims data of HMO to obtain information on actual utilization of healthcare resources. The Short Form Center for Epidemiologic Studies Depression scale was used to assess depressive symptoms. Multivariable logistic regression analyses were conducted to determine predictor variables.

**Results:**

Of 792 respondents, about 17% had depressive symptoms. Almost 96% of patients were using 1 or more antidiabetic medications. Overall, increased risk of depression was associated with lower health related quality of life (HRQoL) (OR: 0.97; 95% CI: 0.96–0.98) and higher impairments in instrumental activities of daily living (IADLs) (OR: 1.31; 95% CI: 1.14–0.52) in elderly patients. Poor health related quality of life (OR: 0.97, 95%CI: 0.95–0.99) was associated with higher risk of depression in patients on insulin therapy.

**Conclusion:**

Impairments in daily activities and lower HRQoL were predictors of depressive symptomatology in elderly with diabetes. Determinants of depression varied according to pharmacotherapeutic class of antidiabetic medications.

## Background

Diabetes affects 20.8 million individuals and is considered as the sixth leading cause of death in the United States [1]. It poses an immense economic burden on the U.S. healthcare system costing around $100 billion annually [1]. Around 7 million elderly Americans suffer from type 2 diabetes mellitus. An elderly population in the United States is associated with maximum utilization of healthcare resources [2].

Like diabetes, depression is growing concern in the United States afflicting around 19 million Americans annually [3]. Of the 35 million Americans of age 65 and older, an estimated two million suffer from depression [4]. The prevalence of depression further increases among the elderly with chronic medical conditions like diabetes. The odds of depression in patients with diabetes are twofold than those without diabetes [5]. Research has reported that approximately 30% of individuals with diabetes have depressive symptomatology and 10% suffer from major depression [6]. Both diabetes and depression were major contributors of functional disability in the elderly population [7]. Furthermore, co-morbid depression in diabetes patients accounted for 4.5 times higher healthcare expenditure than those without depression [8].

Several sociodemographic, clinical and behavioral factors have been considered as determinants of depression in diabetes patients. Studies have shown that age, gender, education and income were significant predictors of depression in diabetic population. [9-11] Depression has been found to be associated with diabetes related psychological and physiological processes including diabetes complication [12], increased blood glucose level [13], and insulin dependence [5]. Behavioral factors such as smoking and alcohol abuse were also associated with depression [14].

The impact of a type of pharmacotherapy on depressive symptomatology in the elderly population with type 2 diabetes has not been studied yet. Patients with diabetes are treated with oral antidiabetic medications (OADs) or insulin or combination therapy depending upon patient characteristics, severity of disease, glycemic level and risk of complications [15]. The impact of pharmacotherapy becomes a major concern in the elderly who are on polypharmacy and highly vulnerable to morbidity and mortality. Hence, the type of anti-diabetic medication therapy may have influence on the comorbid depression in subgroup of patients. Taking into account higher prevalence of depression among elderly, as the baby-boomer generation ages, this illness will contribute to the continued financial strain on the health care system. Thus, the objective of this study was to identify self-reported predictors of depressive symptomatology in the elderly with diabetes.

## Methods

### Study design and population

This was a prospective cohort study started in late 1996 with annual follow up for 2 years postenrollment. The study population consisted of elderly patients (age 65 years or above) with type 2 diabetes mellitus in the southeastern United States who were enrolled in a Medicare Health Maintenance Organization (HMO). This HMO plan was a sole provider of medical care to these enrollees ("lock in" risk benefit plan). The study was conducted by administering a health status assessment questionnaire to nearly 1000 enrollees. The subjects enrolled in Medicare HMO were selected randomly for the purpose of this study and questionnaire was sent to them.

The study was restricted to elderly with type 2 diabetes using some type of pharmacotherapy for diabetes management. The subjects were identified using the codes for type 2 diabetes mellitus (250.xx) from the International Classification of Diseases, Ninth Revision, Clinical Modification (ICD-9-CM) [16] and the HMO's internal medication coding system for prescribing antidiabetic medications.

The survey questionnaires were mailed to participants and informed consent was obtained from them. Participation in this study was voluntary and data were kept confidential. The data were made available to researchers by the Medicare HMO after removal of all patient identifiers. The response rate for the survey was 80%. The questionnaires which provided responses for more than 80% of the items in the study were included in the analysis. All questionnaires were usable. Questionnaire in which depression measure was not reported or incomplete were evaluated for the pattern of missing data. The missing data was random and hence those questionnaires were not included in the study. The study protocol was reviewed and approved by the Institutional Review Board at Wake Forest University School of Medicine for data collection from human subjects. The responses obtained from questionnaire were combined with actual 2-year postenrollment claims data.

### Measurements

The health status assessment instrument collected information regarding demographics, general health status, sedentary lifestyle, functional status, depression, health related quality of life and health care service utilization in preenrollment year. The responses to questionnaire formed basis for the demographic, health status and healthcare utilization variables. The demographic variables were age, gender and whether enrollee lived alone. The health status variables were indicators of sedentary status (from questions examining physical activity and whether the enrollee walked for at least 30 minutes per week), smoking status (i.e. more than 10 cigarettes per day was considered as a heavy smoker) alcohol consumption (i.e. more than 3 drinks per day was considered as a heavy alcohol drinker) and perceived general health status (whether the patient perceived that health status worsened during the year preceding enrollment). Functional status of enrollees was assessed by impairments in the number of activities of daily living (ADLs) (e.g. eating, dressing, taking bath etc.) and the number of instrumental activities of daily living (IADLs) (e.g shopping for grocery, preparing meals etc).

The Medical Outcomes Study Short Form 12 (SF-12) was used to determine health related quality of life (HRQoL) [17]. The Short Form Center for Epidemiologic Studies Depression (CES-D) scale was used to measure depression (dependent variable) on the scale of 0 to 60 [18]. The score of 16 or higher on CES-D was considered as a positive response for depressive symptoms. Funcational status, HRQoL and depression were measured for preenrollment year. Healthcare utilization variables were self-reported prescription medications, physician office visits, emergency room (ER) visits and hospitalizations in the year prior to plan enrollment.

The administrative claims data of patients' HMO was used to retrieve information about actual utilization of healthcare resources during the 2 year postenrollment follow up period. The information obtained was a type of pharmacotherapy, and prescription refills. Responses obtained from survey were combined with actual claim's data acquired from HMO.

### Statistical analysis

Descriptive analyses were performed to evaluate patterns of antidiabetic medication use. Chi square statistics were used to determine differences in categorical variables of diabetic patients with and without depression. An independent sample t test was used for continuous measures. Data were analyzed using STATA statistical package (version 9.1) at a set priori significance level of 0.05.

Multivariate logistic regression model was used to examine associations between predictor variables (as captured by health status assessment questionnaire) and risk of depression. The main model consisted of following predictor variables which are listed as follows: demographics variable consisted of age, gender, health status variables included number of prescriptions, antidiabetic medication use, perceived health status, health related quality of life and self-reported healthcare utilization variables incorporated number of hospitalizations and ER visits in preenrollment year. The regressions were tested for the presence of multicollinearity (ie linear relationship among predictor variables). All correlation coefficients between the predictor variables were less than 0.4 and variation inflation factor of 2 indicated the absence of multicollinearity. Subgroup analyses were performed to identify risk factors of depression in patients with different types of pharmacotherapy.

## Results

The sample consisted of 792 elderly patients with type 2 diabetes mellitus. Almost 60% of the participants were women and mean age of subjects was 71 (± 8.7) years. Of the 792 respondents, about 17% had depressive symptomatology. Most of them (96.7%) were using 1 or more antidiabetic medications. The pharmacotherapy comprised of oral antidiabetic medications, insulin or combination.

Table 1 compares characteristics of individuals with diabetes by depression status. It was found that elderly with depression were mostly of age 70 years and women compared with those without depression (p < 0.05). Patients with depression were more likely to have sedentary lifestyle, poorer general health status, higher impairment in activities of daily living, and lower health related quality of life compared to those without depression (p < 0.05). The elderly with co-morbid depression experienced significantly more number of self-reported hospitalizations and emergency room visits in previous year compared to no depression group (p < 0.05). Subgroup analysis conducted to evaluate the patterns of anti-diabetic medication use indicated that most of the patients (80%) were predominantly using oral antidiabetic medications.

**Table 1:** Summary of descriptive characteristics of the elderly population with type 2 diabetes mellitus and enrolled in a Medicare health maintenance organization

**Characteristic**	**Total (n = 792)**	**With depression (n = 137)**	**Without depression (n = 655)**	**Test statistics (p value)**
Age (years), mean (SD)	71. 95 (8.7)	69.42 (11.33)	72.47 (7.92)	6.3486 (0.000)
Sex (% male)	43.97	39.71	44.87	4.48 (0.034)
Current smokers (%)	12.5	14.59	12.1	2.73 (0.099)
Any alcohol consumption (%)	5.55	3.65	5.95	2.24 (0.134)
Physically active (%)	26.64	16.79	28.70	17.91 (0.000)
SF-12 general health score, mean (SD)	46.09 (25.25)	30.58 (21.82)	49.37 (24.71)	91.29 (0.000)
No of ADLs causing problems, mean (SD)	0.37 (1.07)	0.81 (1.52)	0.28 (0.92)	67.96 (0.000)
No of IADLs causing problems, mean, (SD)	0.97 (1.72)	2.04 (2.24)	0.75 (1.50)	105.05 (0.000)
Prescription refills mean (SD)	3.33 (0.98)	3.62 (0.75)	3.26 (1.01)	0.25 (0.615)
Antidiabetic medication use (%)	99.62	98.54	99.84	0.99 (0.319)
Hospitalization during previous year (%)	29.67	48.9	25.65	25.88 (0.000)
ER visits during previous year (%)	38.28	67.91	32.15	34.91 (0.000)

SF-12 = Medical Outcomes Study 12-item Short Form Health Survey; ADLs = Activities of Daily Living; IADLs = Instrumental Activities of Daily Living; ER = Emergency room.

Independent sample t test was performed for continuous variables such as age, SF-12 scores, No of ADLs causing problems, No of IADLs causing problems, and prescription refills.

Chi^2 ^test of independence was performed for sex, current smoker, any alcohol consumption, physical activities, hospitalization in previous year and ER visits in previous year

Table 2 shows the results of multivariate logistic regression examining predictors of depressive symptomatology. Age was a significant predictor but weakly associated with risk of depression (OR: 0.97; 95% CI: 0.94–0.99). Patients with lower health related quality of life were significantly associated with higher risk of depression (OR: 0.97; 95% CI: 0.96–0.98). Likelihood of depression increased by 31% with higher impairments in instrumental activities of daily living (IADLs) (OR: 1.31; 95% CI: 1.14–0.52).

**Table 2:** Predictors of depression in the elderly population with type 2 diabetes mellitus and enrolled in a Medicare health maintenance organization

	**Depressive symptomatology**			
	
**Independent variables**	**Sample (n = 792)**	**Only insulin (n = 252)**	**Only sulfonylurea (n = 562)**	**Combination therapy (n = 71)**
	
	**Odds ratio (95% CI)**	**Odds ratio (95% CI)**	**Odds ratio (95% CI)**	**Odds ratio (95% CI)**
Age (years)	0.97 (0.94–0.99)*	0.97 (0.94–1.1)	0.97 (0.95–1.00)	0.99 (0.92–1.06)
Male gender	0.67 (.43–1.05)	0.99 (0.47–2.1)	0.48 (0.28–0.82)*	0.62 (0.16–2.36)
Health related quality of life	0.97 (0.96–0.98)*	0.97 (0.95–0.99)*	0.97 (0.96–0.98)*	0.95 (0.91–0.99)*
ADLs causing problems	0.87 (0.71–1.1)	0.83 (0.59–1.12)	0.83 (0.61–1.15)	0.64 (0.23–1.71)
IADLs causing problems	1.31 (1.14–0.52)*	1.24 (1.01–1.53)*	1.29 (1.07–1.56)*	1.31(0.82–2.08)
No of Prescription	1.02 (0.77–1.35)	2.06 (0.90–4.72)	0.91 (0.68–1.23)	0.79 (0.23–2.71)
Hospitalizations during previous year	0.93 (.64–1.28)	1.28 (0.80–2.05)	0.58 (0.35–0.95)*	0.81 (0.31–2.08)
ER visits during previous year	1.22 (0.90–1.66)	1.10 (0.68–1.78)	1.51 (1.04–2.18)*	1.06 (0.49–2.26)
Insulin therapy	0.88 (0.31–2.52)	-	-	-
Sulfonylurea therapy	1.49 (0.55–4.03)	-	-	-
Combination therapy	1.66 (0.54–5.12)	-	-	-

*Statistically significant at p < 0.05.

ADLs = Activities of Daily Living; IADLs = Instrumental Activities of Daily Living; ER = Emergency room.

Table 2 also summarizes the effect of type of antidiabetic therapy on risk of depression. When categorized by pharmacotherapeutic class of antidiabetic medications, poor health related quality of life (OR: 0.97, 95%CI: 0.95–0.99) was associated with higher risk of depression in patients on insulin therapy. Similarly, the odds of depression increases with increased impairments in IADLs. (OR: 1.24, 95%CI: 1.01–1.53) in patients on insulin therapy. However, in patients on sulfonylurea, males were less likely to be at a risk of depression than females (OR: 0.48, 95%CL: 0.28–0.82). Likelihood of depression was associated with self-reported hospitalization (OR: 0.55, 95%CI: 0.34–0.88), and emergency room visits in previous year (OR: 1.45, 95%CI: 1.01–2.08). The odds of depression increases with lower health related quality of life (OR: 0.97, 95%CI: 0.96–0.98) and with increased impairments in IADLs (OR: 1.29, 95%CI: 1.07–1.56) in patients on sulfonylurea therapy. When patients on combination therapy were examined, likelihood of depression was associated with lower health related quality of life (OR: 0.95, 95%CI: 0.91–0.99).

## Discussion

This study revealed that co-morbid depression in elderly with diabetes was associated with younger age, lower health related quality of life and higher impairment in instrumental activities of daily living. Our findings are consistent with the existing literature [19-24]. SF-12 could be used as an effective tool to detect depression and demonstrate health related quality of life in diabetic patients with depression. The Diabetes-Specific Quality of Life Scale will be also helpful to measure depression in these patients. Periodical assessment of patients' health related quality of life can give an early opportunity to health care professionals and hospital administrators to identify patients at higher risk of depression and refer them to proper disease risk management program. Similarly, the odds of depressive symptoms were significantly high in the elderly with higher impairment in instrumental activities of daily living. IADL limitations are also attributed to physical health related chronic conditions co-morbidity such as diabetes [22]. The literature has documented that impaired activities in diabetic patients can cause substantial problems such as functional decline, dependency and physical disability [7,22,23]. Hence, the functional status of elderly with diabetes can be considered as an important indicator for identifying patient at a risk of depression.

This study also examined the impact of prescribing pharmacotherapy on risk of depression in diabetic patients. Interestingly, the pharmacotherapeutic class of antidiabetic medications has a significant impact on the risk of depression in diabetic patients. Patients with type 2 diabetes who fail to respond adequately to oral antidiabetic medications (OADs) or whose glycemic control worsens despite using recommended combinations of OADs often start insulin therapy [25]. The study found that among the patients on insulin therapy, likelihood of depression increased with the increased impairments in IADLs. Among the patients on insulin therapy and/or combination therapy, higher odds of depression were associated with lower HRQoL as measured by SF-12 instrument. When we examined elderly patients on sulfonylurea therapy, likelihood of comorbid depression increases in females compared to males. Similar to other antidiabetic medications, the odds of depression were associated with lower HRQoL and increased impairment in IADLs. The odds of self reported hospitalizations were lower in patients on sulfonylurea therapy in previous year. It may be due to lower number of complications associated with use of sulfonylurea in elderly patients. However, this conclusion can not be confirmed from the given analysis.

These findings have huge implications from public health perspective. Monitoring patients' characteristics, anti-diabetic therapy and medication use patterns will facilitate early identification of patients at risk of depression. Patients on insulin therapy commonly face problems such as needle anxiety, fear of injection pain, and inconvenience of administration, troublesome dosing schedule, and social stigma [26-28]. Patient's psychological and behavioral characteristics may be associated with poor HRQoL and associated depression. Hence, clinicians should take special efforts in educating and counseling patients regarding appropriate use of insulin therapy. This study also support and emphasizes an importance of developing patient-centered pharmacotherapy to preclude the problems associated with depression. It is essential for policy makers to develop special screening guidelines for depression in a subset of patients on sulfonylurea therapy. Especially female patients using sulfonylurea with higher frequency of ER visits should be targeted for early screening and monitoring of depression.

The results of this study should be generalized with caution since data were obtained from single geographically located Medicare HMO. The study could not evaluate the effect of risk factors such as glycemic control, social support on depressive symptomatology due to lack of availability of data. Additionally, the observational study design does not permit causal inference between diabetes and depression.

This study strengthens the hypothesis that there is a strong relationship between depression and diabetes. Depression may exacerbate, or alternatively be exacerbated by diabetes. Diabetes management program can substantially benefit elderly who are at risk of depression in terms of improving health outcomes and quality of life. Taking into account a wide variation in the treatment pattern and its impact on comorbid depression, hospital administrators should focus on patient centered pharmacotherapy, timely screening and improving patients' health status.

## Conclusion

Comorbid depression is prevalent in elderly patients with type 2 diabetes mellitus. Patient characteristics such as age, lower health related quality of life and impairment in instrumental activities of daily livings were significant determinants of depression in diabetic patients. Type of pharmacotherapeutic class of anti-diabetic medications has a significant impact in predicting comorbid depression in elderly population. Hence, health care professionals should take into consideration likelihood of comorbid depression while prescribing pharmacotherapy for the treatment of type 2 diabetes.

## Competing interests

This study was not funded by any source. Programming assistance provided by Fabian Camacho is acknowledged.

## Authors' contributions

MP carried out extensive literature review, analysis and interpretation of data; she has been involved in drafting the manuscript and revising it.

RA involved in conceptualization and designing a study. He has provided valuable insights for revising manuscript.

RB conceived of the study, and participated in its design and coordination and helped to draft the manuscript.

All authors read and approved the final manuscript.
